# Variations in CD14 Gene Are Associated With Autoimmune Thyroid Diseases in the Chinese Population

**DOI:** 10.3389/fendo.2018.00811

**Published:** 2019-01-16

**Authors:** Xi Jia, Bing Wang, Qiuming Yao, Qian Li, Jinan Zhang

**Affiliations:** ^1^Department of Endocrinology, Jinshan Hospital of Fudan University, Shanghai, China; ^2^Department of Endocrinology, Shanghai University of Medicine and Health Sciences Affiliated Zhoupu Hospital, Shanghai, China

**Keywords:** CD14, single nucleotide polymorphisms, autoimmune thyroid diseases, Graves' disease, Hashimoto's thyroiditis

## Abstract

Autoimmune thyroid diseases (AITDs) are chronic organ-specific autoimmune diseases and mainly include Graves' disease (GD) and Hashimoto's thyroiditis (HT). CD14 is an important component of the immune system as a receptor for gram-negative lipopolysaccharide (LPS). The genetic polymorphisms of CD14 have been confirmed to be associated with a variety of autoimmune diseases. However, its relationship with AITDs is still unclear. The study was aimed to determine whether four single nucleotide polymorphisms (rs2915863, rs2569190, rs2569192, and rs2563298) of CD14 are associated with AITDs and its subgroups of GD and HT. The results showed significant association of rs2915863 and rs2569190 with GD. The frequencies of rs2915863 genotypes and T allele in patients with GD differed significantly from their controls (*P* = 0.007 and *P* = 0.021, respectively). For rs2569190, frequencies of genotypes and G allele in GD patients also showed positive *P*-values (*P* = 0.038 and *P* = 0.027, respectively). The correlations between these two loci and GD are more pronounced in female GD patients and patients with a family history. In genetic model analysis, the allele model, recessive model, and homozygous model of rs2569190 and rs2915863 embodied strong correlations with GD after the adjusting of age and gender (*P* = 0.014, *P* = 0.015, *P* = 0.009, and *P* = 0.014, *P* = 0.001, *P* = 0.006, respectively). However, these four sites are not related to HT. We firstly discovered the relationship between CD14 gene polymorphism and GD, and the results indicate that CD14 is an important risk locus for AITD and its SNPs may contribute to host's genetic predisposition to GD.

## Introduction

Autoimmune thyroid diseases (AITDs) are chronic thyroid-specific autoimmune diseases and mainly include Graves' disease (GD) and Hashimoto's thyroiditis (HT) (1). The prevalence of AITDs is estimated to be 5% in general population and 5–10 times in women than in men (2, 3). The specific pathogeny of AITDs is still unclear and may be related to genetic susceptibility, immunopathogenic mechanisms, and environmental factors (4, 5).

The cluster differentiation antigen 14 (CD14) gene is localized on chromosome 5q31.1 region and encodes a glycosylphosphatidylinositol-anchored membrane glycoprotein (6). As a pattern recognition factor, CD14 protein is constitutively expressed in majority of innate immune response cells, and plays a central role in innate immunity through recognition of bacterial lipopolysaccharide (LPS) (6, 7). CD14 protein exists mainly in membrane form (mCD14) or soluble form (sCD14) (8). Membrane CD14 is expressed primarily on the surface of monocytes, macrophages, and neutrophils, while sCD14 is predominantly in serum (8). Detection of sCD14 in serum can partially reflect the expression of CD14 gene *in vivo*. As an important component of innate immunity, alterations in CD14 expression appear to correlate with aberrant immune responses and autoimmune diseases. The role of CD14 polymorphisms in autoimmune disorders has been widely explored, including inflammatory bowel disease (IBD) (9–11), multiple sclerosis (MS) (12), rheumatoid arthritis (RA) (13–15), juvenile idiopathic arthritis (16), systemic lupus erythematosus (15), and type 1 diabetes mellitus (T1DM) (17). Different autoimmune diseases often share some common immunological mechanisms. Therefore, it is reasonable to speculate that CD14 polymorphisms may contribute to AITDs. This study was conducted to explore the association of four CD14 polymorphisms (rs2915863, rs2569190, rs2569192, and rs2563298) with the AITDs in the population of south China, and to explore its mechanisms though bioinformatics analysis.

## Materials and Methods

### Patients and Healthy Individuals

In our study, we conduct an anonymized cohort involving 847 Chinese Han AITDs patients and 715 healthy Chinese Han controls. The AITDs patients included 522 GD patients (363 males and 159 females) and 325 HT patients (49 males and 276 females). The male to female ratio of our sample is in consistent with that in general population. In order to exclude sampling bias, all of them were randomly recruited individuals living in the same geographic region (Shanghai, China), without any genetic relationship. All AITDs patients were recruited from the Out-patient Department of Endocrinology of Jinshan Hospital. Healthy controls were consecutively enrolled from the Healthy Check-Up Center of the same hospital with ethnically and geographically matching. The control group participating in the study did not have any history of immune diseases or other chronic diseases. The study was approved by the ethical committees of Jinshan Hospital. All enroll individuals in the AITDs group and the control group provided verbal and written informed consent.

The diagnosis criteria of GD used in this study included clinical manifestations of thyrotoxicosis, biochemical indicators of hyperthyroidism, positive circulating thyroid-stimulating hormone receptor antibody (TRAb) and diffuse goiter of the thyroid observed by B-ultrasound or palpation. HT cases were defined on the basis of enlarged thyroid and elevated level of either thyroid peroxidase antibody (TPOAb) or thyroglobulin antibody (TgAb).

### DNA Sample Collection and Extraction

Genomic DNA was extracted from 2 ml peripheral venous blood of each participants using the Relax Gene Blood DNA System (Tiangen Biotech Co., Ltd., Beijing, China). The concentration and purity of DNA was measured using Nano Drop 2,000 Spectra-photometer (Thermo Scientific Company, Waltham, MA, USA).

### SNP Selection and Genotyping

Four SNPs of CD14 were investigated in the present study, including rs2915863, rs2569190, rs2569192, and rs2563298. In the light of previously published literature, significant associations of these loci with multiple autoimmune diseases have been identified. Therefore, there have a theoretical basis for us to speculate that they may also have correlations with the susceptibility of AITDs. The Hardy-Weinberg equilibrium *P*-values (HWpval) of these four SNPs met the criteria of HWpval > 0.05. The target DNA sequences were amplified by multiplex polymerase chain reaction (PCR) method using specific primers with sequences shown in Table 1.

**Table 1:** specific primer sequences of SNPs.

**SNPs**	**Primer**
rs2915863	Forward Primer- TCTCAAAGTGCTGGGATTACAG Reverse Primer- AAATACAAAATTAGCCGGGTGTAG
rs2569190	Forward Primer- CCTCTGTGAACCCTGATCACCTCC Reverse Primer- CGCCTGAGTCATCAGGACACTGC
rs2569192	Forward Primer- ACTCACAGCTTGATTCAACAAATG Reverse Primer- TTGGTTTCTCTTCTTTTAAGAGCC
rs2563298	Forward Primer- GATAGGGTTTCTTAGGGAGTTAGG Reverse Primer- AATAATGAATGGACTCAAACTGCC

### Genotyping-Clinical Phenotype Analysis

Different clinical manifestations may have different genetic backgrounds. In order to more accurately investigate the relationship between SNPs and different clinical phenotypes of AITDs, the clinical classifications of GD and HT in the current study were set as (i) presence or absence of thyrotoxic exophthalmos in the GD group; (ii) the thyroid goiter degree or normal volume; and (iii) presence or absence of AITD family history (disease in the first-degree relatives). The characteristics of all experimental subjects are summarized in Table 2.

**Table 2:** Demographic statistics and clinical phenotypes of subjects in case group.

	**AITDs (%)**	**GD (%)**	**HT (%)**
Number	847	522	325
**GENDER**			
Female	639 (75.44)	363 (69.54)	276 (84.92)
Male	208 (24.56)	159 (30.46)	49 (15.08)
Age	41.87 ± 14.53	41.39 ± 14.79	42.60 ± 14.06
Ophthalmopathy (+)	86 (10.15)	83 (15.90)	3 (0.92)
Family history (+)	162 (19.13)	108 (20.69)	54 (16.62)

*AITDs, autoimmune thyroid diseases; GD, Graves' disease; HT, Hashimoto's thyroiditis*.

Graves' ophthalmopathy (GO), also called thyroid-associated opthalmopathy, is a common extra-thyroid manifestation of GD, mainly manifested as inflammation and swelling of the extraocular muscles, chemosis, eyelid edema, proptosis, excess tearing, and episcleral vascular injection (1, 18). Graves' ophthalmopathy (GO) was diagnosed by the clinical assessment criteria for GO from Williams Textbook of Endocrinology (19).

### Statistical Analysis

All odds ratios (OR), 95% confidence interval (95%CI), and *P*-values were calculated using the Stata version 12.1 software (Stata, Inc.), based on the two-tailed Pearson chi-square test (*X*^2^ test) for genotype/allele frequency of each SNP. *P* < 0.05 was considered statistically significant. For each SNP, deviation from Hardy-Weinberg equilibrium (HWE) was estimated using the HWE program (http://ihg.gsf.de/cgi-bin/hw/hwa1.pl) in controls and cases separately. Linkage analysis and haplotype analysis were also performed in this study. A linkage disequilibrium (LD) test was conducted using Haploview Software (version 4.2, Broad Institute, Cambridge, MA, USA). To consolidate the evidence, significant findings were further examined by multiple logistic regression (Stata 12.1, Inc.) and adjusted for potential interfering factors (gender and age) simultaneously.

### Bioinformatics Analysis

#### Associations of CD14 Expression Level With Key Immune Cells in GD Tissues

The correlations of CD14 expression level with key immune cells in 18 GD thyroid tissues were studied through using GSE9340 from Gene Expression Omnibus (GEO) database (20). Macrophages, plasma B cells, T follicular helper cells (Tfh) and regulatory T cells (Tregs) in GD tissues were estimated from the gene expression profiles in GSE9340 by CIBERSORT tool (21). Th1 and Th2 in D tissues were estimated from the gene expression profiles in GSE9340 by Cell tool (22). To further assess the roles of CD14 in GD, the correlations of its expression and intrathyroidal immune cells were analyzed using Spearson correlation analysis.

#### Functional Pathways Related to CD14 in GD Tissues

Gene set enrichment analysis (GSEA) was done to identify crucial functional pathways related to CD14 through using gene expression profiling of 18 GD tissues from GSE9340 (23). GSEA analysis was performed with GSEA v3.0, and GO biological process (4,436 genes sets) were used as predefined genes sets. Gene sets with both an Enrichment score (ES) more than 0.70 and false discovery rate (FDR) *q* < 0.05 were considered significantly enriched pathways.

## Results

### Association of CD14 rs2915863 and rs2569190 With GD

In the current study, we examined the frequency distribution for each allele and analyzed the association for each SNP in a case-control manner. Associations of SNPs in CD14 gene with AITDs, GD and HT are shown in Table 3. Although there are no significant association between these four SNPs (rs2915863, rs2569190, rs2569192, and rs2563298) and AITDs, rs2915863 and rs2569190 are significantly correlated with GD. Both genotyping and allele analyses of rs2915863 showed significant *P*-values (P_genetyping_ = 0.007 and P_allele_ = 0.021, respectively). Moreover, rs2569190 also obtained similar results, and the *P*-values of the genotyping and allele analyses were 0.038 and 0.027.

**Table 3:** Associations of rs2915863, rs2569190, rs2569192, and rs2563298 in CD14 gene with AITDs, GD, and HT.

**SNP**	**Genotype or allele**	**NC**	**AITD**	***P*-value**	**GD**	***P*-value**	**HT**	***P*-value**
		***n* (%)**	***n* (%)**	**AITD vs. NC**	***n* (%)**	**GD vs. NC**	***n* (%)**	**HT vs. NC**
rs2915863	CC	225 (31.47)	258 (30.46)	0.060	153 (29.31)	**0.007**	105 (32.31)	0.889
	TC	370 (51.75)	407 (48.05)		244 (46.74)		163 (50.15)	
	TT	120 (16.78)	182 (21.49)		125 (23.95)		57 (17.54)	
	C	820 (57.34)	923 (54.49)	0.109	550 (52.68)	**0.021**	373 (57.38)	0.986
	T	610 (42.66)	771 (45.51)		494 (47.32)		277 (42.62)	
rs2569190	AA	270 (37.76)	311 (36.72)	0.109	178 (34.10)	**0.038**	133 (40.92)	0.358
	AG	353 (49.37)	395 (46.64)		250 (47.89)		145 (44.62)	
	GG	92 (12.87)	141 (16.65)		94 (18.01)		47 (14.46)	
	A	893 (62.45)	1,017 (60.04)	0.168	606 (58.05)	**0.027**	411 (63.23)	0.732
	G	537 (37.55)	677 (39.96)		438 (41.95)		239 (36.77)	
rs2569192	GG	554 (77.48)	650 (76.74)	0.929	396 (75.86)	0.775	254 (78.15)	0.784
	CG	155 (21.68)	189 (22.31)		122 (23.37)		67 (20.2)	
	CC	6 (0.84)	8 (0.94)		4 (0.77)		4 (1.23)	
	G	1,263 (88.32)	1,489 (87.90)	0.716	914 (87.55)	0.559	575 (88.46)	0.927
	C	167 (11.68)	205 (12.10)		130 (12.45)		75 (11.54)	
rs2563298	CC	552 (77.20)	649 (76.62)	0.955	396 (75.86)	0.866	253 (77.85)	0.695
	AC	158 (22.10)	190 (22.43)		122 (23.37)		68 (20.92)	
	AA	5 (0.70)	8 (0.94)		4 (0.77)		4 (1.23)	
	C	1,262 (88.25)	1,488 (87.84)	0.841	914 (87.55)	0.675	574 (88.31)	0.846
	A	168 (11.75)	206 (12.16)		130 (12.45)		76 (11.69)	

*AITDs, autoimmune thyroid diseases; GD, Graves' disease; HT, Hashimoto's thyroiditis; NC, Normal Controls. Bold represents a positive P value*.

Since rs2915863 and rs2569190 are significantly associated with GD and different clinical subpopulations may have different genetic backgrounds, we further analyzed the gender and family history subgroups of GD patients. As shown in Table 4, for rs2915863, female GD patients show a stronger positive *P*-value (P_genetyping_ = 0.005, P_allele_ = 0.000) than GD patients in general population (P_genetyping_ = 0.007, P_allele_ = 0.021). Similarly, rs2569190 also has stronger association with female GD patients (*P* = 0.026) as well as GD patients with a positive family history (*P* = 0.011) than in general GD patients (*P* = 0.038). Moreover, this difference is more pronounced in the allele analysis. The *P*-values for female GD patients and GD patients with family history are 0.015 and 0.004, respectively, much more significant than that for GD patients in general population (*P* = 0.027).

**Table 4:** Associations of rs2915863 and rs2569190 in CD14 gene with female GD patients and GD patients with family history.

**SNP**	**Genotype or allele**	**NC**	**Female GD patients**	***P*-value**	**GD with family history**	***P*-value**
		***n* (%)**	***n* (%)**		***n* (%)**	
rs2915863	CC	225 (31.47)	108 (29.75)	0.005	25 (23.15)	0.024
	TC	370 (51.75)	164 (45.18)		54 (50.00)	
	TT	120 (16.78)	91 (25.07)		29 (26.85)	
	C	820 (57.34)	307 (42.29)	0.000	104 (48.15)	0.011
	T	610 (42.66)	419 (57.71)		112 (51.85)	
rs2569190	AA	270 (37.76)	119 (32.78)	0.026	29 (26.85)	0.011
	AG	353 (49.37)	176 (48.48)		55 (50.93)	
	GG	92 (12.87)	68 (18.73)		24 (22.22)	
	A	893 (62.45)	414 (57.02)	0.015	113 (52.31)	0.004
	G	537 (37.55)	312 (42.98)		103 (47.69)	

*AITDs, autoimmune thyroid diseases; GD, Graves' disease; HT, Hashimoto's thyroiditis; NC, Normal Controls*.

To further understand the roles of rs2915863 and rs2569190 in GD, we analyzed the relationship between SNPs and GD in different genetic models, as shown in which Tables 5, 6, correspondingly. In Table 5, we can see that in the model analysis of CD14 and AITDs, only the recessive model of rs2915863 has obvious positive results, and the *P*-values before and after correction are 0.019 and 0.012, respectively. It is clear from Table 6 that rs2915863 has strong association with GD in the allele model, recessive model and homozygous model before (*P* = 0.013, *P* = 0.001, and *P* = 0.006, respectively) and even after (*P* = 0.014, *P* = 0.001, and *P* = 0.006, respectively) adjustment for possible cofounders (age and gender) and rs2569190 also display strong correlations with GD in the allele model, recessive model and homozygous model before (*P* = 0.012, *P* = 0.015, and *P* = 0.008, respectively) and after (*P* = 0.014, *P* = 0.015, and *P* = 0.009, respectively) adjustment for the possible cofounders (age and gender). Furthermore, both rs2915863 and rs2569190 are not related to HT (*P* > 0.05), and both rs2569192 and rs2563298 of CD14 are not related to AITDs, GD, and HT.

**Table 5:** Odds ratios (ORs) of the associations of polymorphisms in CD14 gene with AITD before and after adjustment for confounders (age and gender).

**Comparison models**	**Unadjusted estimates**		**Adjusted estimates**	
	**OR (95%CI)**	***P*-values**	**OR (95%CI)**	***P*-values**
**rs2915863**				
Allele model	1.12 (0.97–1.29)	0.108	1.13 (0.98–1.31)	0.098
Dominant model	1.05 (0.85–1.30)	0.668	1.04 (0.84–1.30)	0.714
Recessive model	1.36 (1.05–1.75)	**0.019**	1.40 (1.07–1.81)	**0.012**
Homozygous model	1.15 (0.99–1.33)	0.060	1.16 (1.1–1.35)	0.050
Additive model	0.96 (0.76–1.20)	0.720	0.94 (0.75–1.19)	0.618
**rs2569190**				
Allele model	1.10 (0.95–1.28)	0.183	1.12 (0.98–1.28)	0.255
Dominant model	1.03 (0.83–1.26)	0.810	1.03 (0.83–1.27)	0.807
Recessive model	1.33 (1.00–1.78)	0.048	1.32 (0.99–1.77)	0.063
Homozygous model	1.15 (0.99–1.35)	0.070	1.15 (0.99–1.35)	0.075
Additive model	0.97 (0.78–1.21)	0.794	0.98 (0.79–1.22)	0.863
**rs2569192**				
Allele model	1.04 (0.82–1.32)	0.709	1.02 (0.81–1.28)	0.857
Dominant model	1.04 (0.55–1.60)	0.728	1.02 (0.80–1.30)	0.879
Recessive model	1.13 (0.39–3.26)	0.826	1.10 (0.37–3.23)	0.863
**rs2563298**				
Allele model	1.04 (0.83–1.30)	0.716	1.02 (0.81–1.28)	0.888
Dominant model	1.03 (0.82–1.31)	0.787	1.00 (0.79–1.28)	0.973
Recessive model	1.35 (0.44–4.15)	0.596	1.36 (0.44–4.24)	0.597

*Allele model = G vs. C; Dominant model = (GG+GC) vs. CC; Recessive model = GG vs. (GC+CC); Homozygous model = GG vs. CC; Additive model = GC vs. CC. Bold represents a positive P value*.

**Table 6:** Odds ratios (ORs) of the associations of four polymorphisms in the CD14 gene with GD before and after adjusting for confounders (age and gender).

**Comparison models**	**Unadjusted estimates**		**Adjusted estimates**	
	**OR(95%CI)**	***P*-values**	**OR (95%CI)**	***P*-values**
**rs2915863**				
Allele model	1.21 (1.04–1.41)	**0.013**	1.21 (1.04–1.40)	**0.014**
Dominant model	1.12 (0.89–1.41)	0.329	1.11 (0.88–1.39)	0.391
Recessive model	1.53 (1.18–1.98)	**0.001**	1.55 (1.20–2.00)	**0.001**
Homozygous model	1.23 (1.06–1.43)	**0.006**	1.23 (1.06–1.43)	**0.006**
Additive model	0.99 (0.77–1.26)	0.919	0.97 (0.76–1.24)	0.791
**rs2569190**				
Allele model	1.21 (1.04–1.42)	**0.012**	1.21 (1.04–1.41)	**0.014**
Dominant model	1.22 (0.98–1.52)	0.073	1.21 (0.97–1.51)	0.089
Recessive model	1.42 (1.07–1.89)	**0.015**	1.42 (1.07–1.90)	**0.015**
Homozygous model	1.24 (1.06–1.45)	**0.008**	1.23 (1.05–1.45)	**0.009**
Additive model	1.13 (0.90–1.43)	0.281	1.12 (0.89–1.42)	0.335
**rs2569192**				
Allele model	1.08 (0.86–1.37)	0.495	1.08 (0.86–1.37)	0.501
Dominant model	1.11 (0.86–1.42)	0.417	1.11 (0.86–1.42)	0.424
Recessive model	0.80 (0.25–2.55)	0.700	0.80 (0.25–2.57)	0.709
**rs2563298**				
Allele model	1.07 (0.85–1.36)	0.546	1.07 (0.85–1.36)	0.557
Dominant model	1.08 (0.85–1.39)	0.495	1.09 (0.85–1.40)	0.509
Recessive model	0.88 (0.27–2.89)	0.839	0.90 (0.27–2.93)	0.855

*Allele model = G vs. C; Dominant model = (GG+GC) vs. CC; Recessive model = GG vs. (GC+CC); Homozygous model = GG vs. CC; Additive model = GC vs. CC. Bold represents a positive P value*.

### Correlations of CD14 With Intrathyroidal Immune Cells

**Figure 1:**
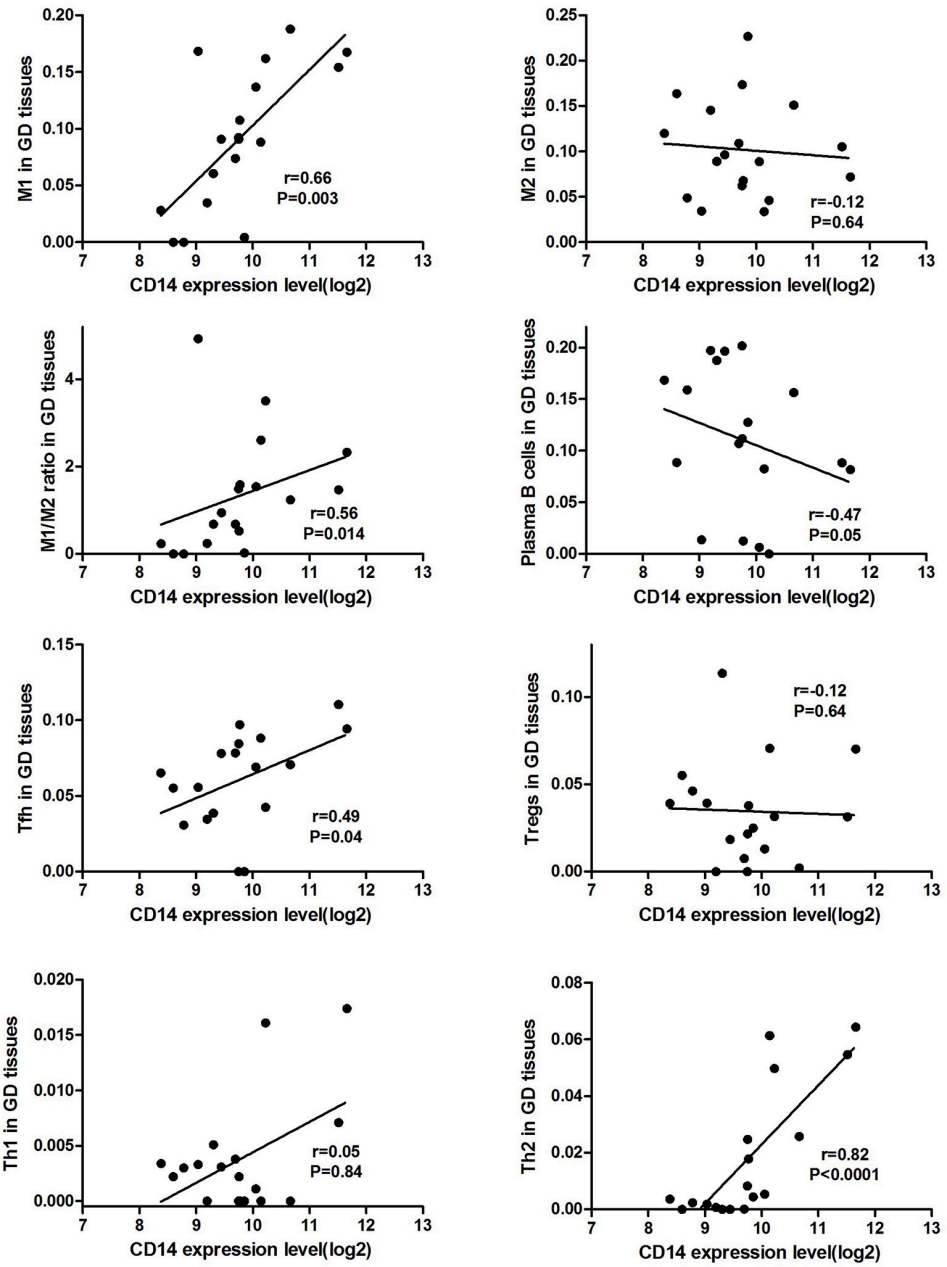
Correlations of CD14 with key intrathyroidal immune cells in GD tissues.

CD14 expression level was positively correlated the proportion of M1 cell (*r* = 0.66, *P* = 0.003) and M1/M2 ratio (*r* = 0.56, *P* = 0.014) in GD tissues (Figure 1). Additionally, CD14 expression level was also positively correlated with the proportion of Tfh cell (*r* = 0.49, *P* = 0.04) and Th2 (*r* = 0.82, *P* < 0.0001) in GD tissues (Figure 1). CD14 was not correlated with the proportion of Th1 cell (*r* = 0.05, *P* = 0.84) in GD tissues (Figure 1).

### Functional Pathways Related to CD14 in GD Pathogenesis

**Figure 2:**
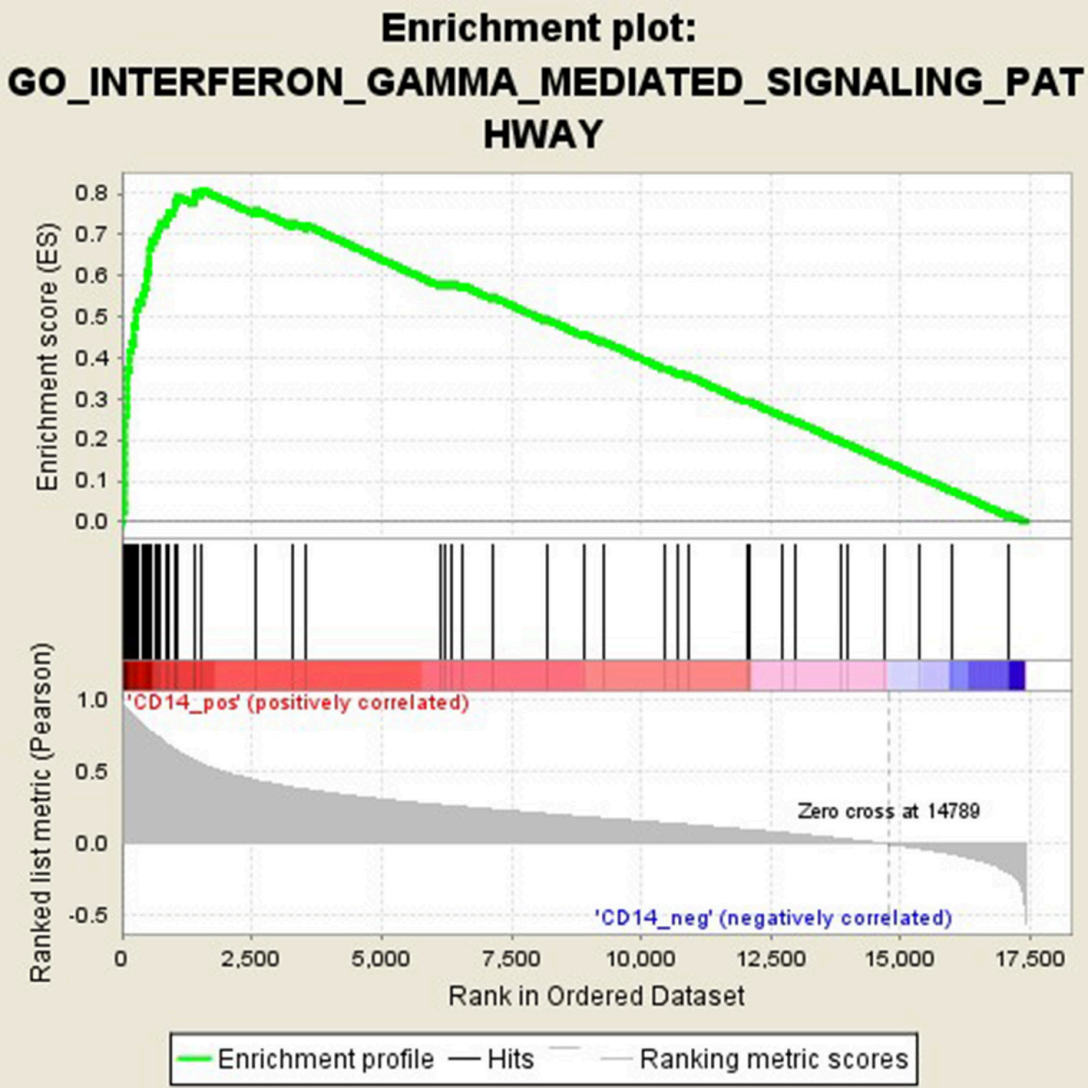
Enrichment plot for the gene set of “Interferon-gamma-mediated signaling pathway”.

**Figure 3:**
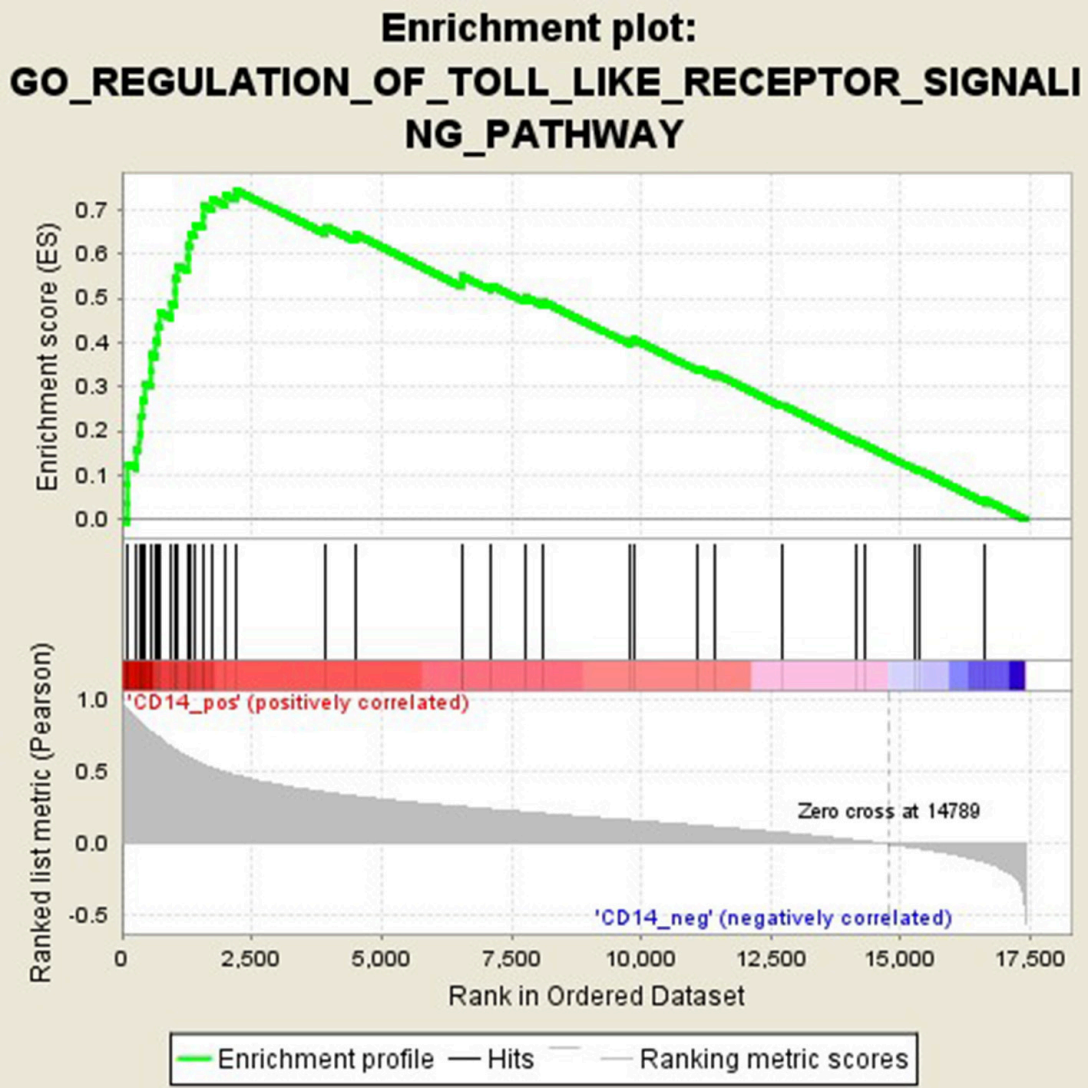
Enrichment plot for the gene set of “Regulation of toll-like receptor signaling pathway”.

**Figure 4:**
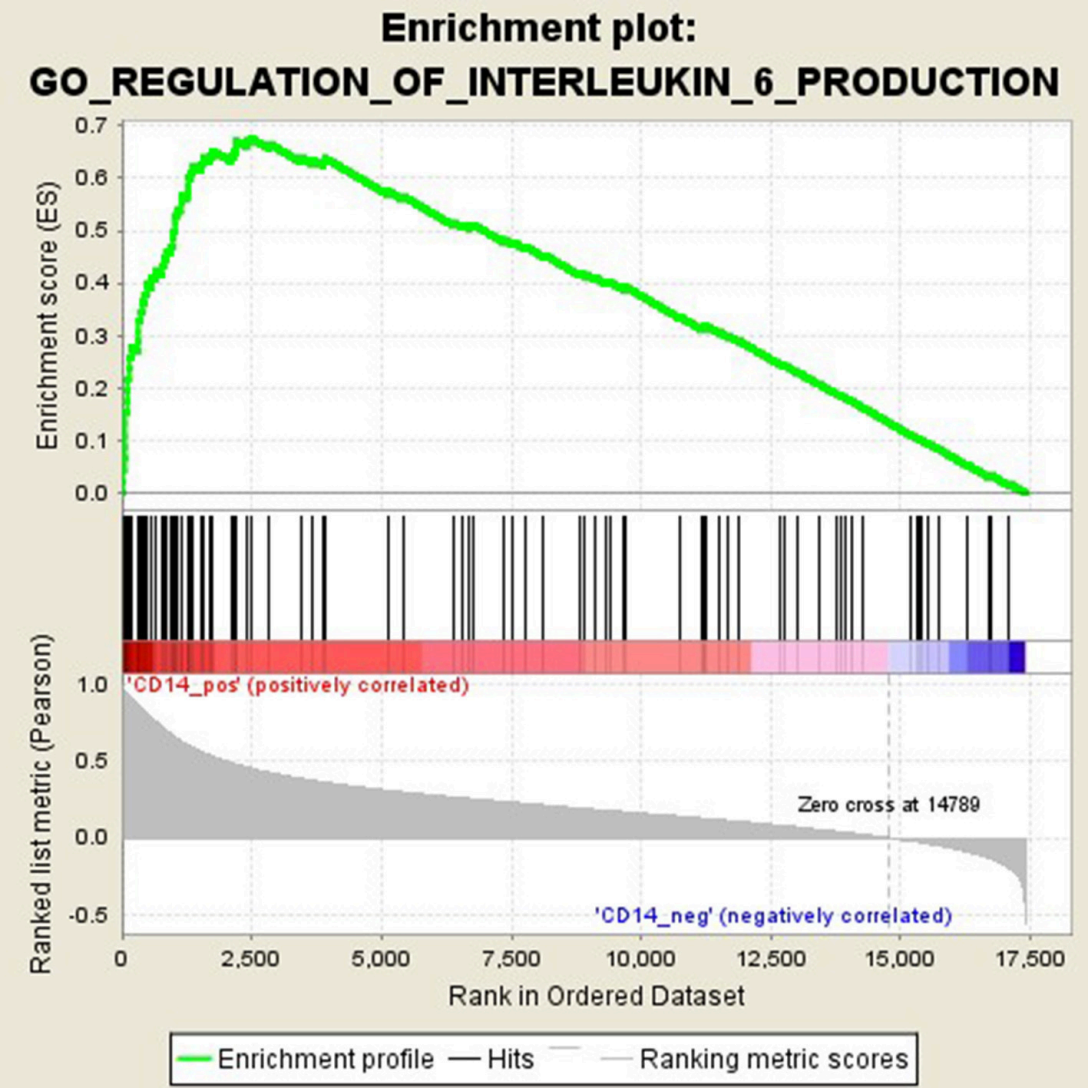
Enrichment plot for the gene set of “Positive regulation of interleukin-6 production”.

GSEA analysis suggested that there were a total of 93 significantly enriched pathways related to CD14 in GD tissues (Supplementary Table 1). Most of those enriched pathways were immunity-related pathways, suggesting the critical role of CD14 in the development of GD through regulating immune response. Table 7 showed the top 10 significantly enriched pathways in the GSEA analysis, such as Interferon-gamma-mediated signaling pathway, Regulation of toll-like receptor signaling pathway, and Positive regulation of interleukin-6 production (Figures 2–4, Table 7).

**Table 7:** Top 10 significantly enriched pathways in the GSEA analysis.

**Gene set**	**ES**	**Nominal *P*-value**	**FDR *q*-value**
Negative regulation of lipid catabolic process	0.84	< 0.001	0.002
Interferon-gamma-mediated signaling pathway	0.81	0.002	0.022
Response to interferon-gamma	0.73	0.004	0.015
Regulation of toll-like receptor signaling pathway	0.75	0.002	0.016
High-density lipoprotein particle remodeling	0.78	< 0.001	0.016
Cellular response to interferon-gamma	0.72	0.006	0.014
Regulation of mast cell activation	0.74	< 0.001	0.013
Positive regulation of interleukin-6 production	0.70	0.002	0.012
Regulation of natural killer cell mediated immunity	0.75	0.006	0.014
Positive regulation of toll-like receptor signaling pathway	0.83	0.002	0.014

*ES, Enrichment score; FDR, false discovery rate*.

## Discussion

CD14 is a receptor that recognizes LPS and other bacterial wall components (8). In CD14 gene, rs2915863 or CD14/−1720 is located in 5' near gene region, while rs2569190, also known as CD14/−260 or C(−159)T, is located in the promoter region (24). Both rs2915863 and rs2569190 can affect the expression level of CD14 *in vivo*, supported by the fact that they are associated with altered levels of sCD14 (25–29). sCD14 is an acute phase protein mainly produced by liver, and can reflect inflammatory disease activity (30). In the present study, we found that two variants rs2915863 and rs2569190 in CD14 gene show significant associations with GD, especially for females GD patient and GD patients with family history. The frequencies of rs2915863 minor allele T and rs2569190 minor allele G are greatly increased in patients with GD. Genetic model analysis shows that rs2915863 and rs2569190 have strong correlations with GD in allele model, recessive model and homozygous model, suggesting that polymorphisms rs2915863 and rs2569190 of CD14 gene may be contributors to the causes of GD.

AITDs are classic autoimmune diseases, with multiple factors involved. AITDs have been confirmed to be associated with SNPs of various immune genes, including CD40, CTLA4, and IL-21 and so on (31–33). Since autoimmune diseases have a certain degree of similarity in immune imbalance and genetic background, a SNP is often found to be associated with multiple autoimmune diseases. Previous studies have found that rs2915863 is associated with symptomatic airway hyper-responsiveness, which may be related to endotoxin exposure and elevated IgE (34–36), and rs2569190 may contribute to allergic rhinitis (37), allergic asthma (24), and IBD (11). In this study, we, for the first time, found that rs2915863 and rs2569190 are significantly related to GD, especially in female patients. Our findings that the role of rs2915863 and rs2569190 in promoting GD are more pronounced in female patients is consistent with the universal acceptance that there is a gender difference in the incidence of GD. The mechanism of preponderance of females in GD has not yet been fully elucidated, and may be related to sex hormones, genetic susceptibility and mental factors (3, 38). Although GD is not a genetic disease in the traditional sense, it has a certain degree of genetic predisposition (2). The family aggregation of GD supports the importance of genetic factors in its pathogenesis. Moreover, our study found that the relationship between CD14 polymorphisms and GD is more pronounced in patients with family history. Compared with the general GD group, the susceptible allele T in rs2915863 and the susceptible allele G in rs2569190 have stronger positive *P*-values in patients with family history. Thus, mutations in the rs2915863 and rs2569190 loci of CD14 gene may have family aggregation and may play a greater role in GD patients with family history.

There is sufficient evidence to support that mutations at these two sites can cause changes in CD14 expression levels and methylation levels, suggesting that they can affect CD14 function (39–41). As an important component of the innate immune system, CD14 has been demonstrated to be involved in infectious and immune-related diseases (42, 43). Membrane CD14 is mainly expressed on the surface of monocyte macrophages, and is one of the specific molecular markers of monocytes/macrophages (44, 45). Macrophages are important antigen-presenting cells (APCs) that mediate homeostasis of the immune system and can currently be divided into two subgroups, M1 and M2 cells (44, 46). CD14, LPS, and LPS binding proteins bind to form a ligand complex that is recognized by the Toll-like receptor-4 (TLR-4)/MD-2 receptor, mediates antigen presentation, stimulates T cell activation and promotes B cell production of antibodies (43, 47). AITDs are chronic organ-specific autoimmune diseases characterized by the production of autoimmune antibodies that attack thyroid cells (1). A variety of immune cells mediate the pathogenesis of AITDs, and macrophages in which membrane CD14 is located are one of the hotspots of recent research. We found through bioinformatics analysis that CD14 expression level was positively correlated the proportion of macrophages M1 cell and M1/M2 ratio in GD tissues (Figure 1), suggesting that CD14 is an important molecule mediating macrophage homeostasis in thyroid tissue of patients with GD. Excessive activation of macrophages, especially its M1 subtype, is closely related to the occurrence of autoimmune diseases, including systemic lupus erythematosus, inflammatory bowel disease, rheumatoid arthritis, and multiple sclerosis (46, 48–53). In GD, infiltrating macrophages were observed in thyroid tissue of AITD patients and in the periorbital tissues of patients with GO (54–56). Infiltrating macrophages can directly destroy thyroid follicular cells (57) or kill thyroid cells through the autologous apoptosis pathway (58).

Activation of T cells is also one of the diverse immune functions of CD14. CD14 can promote the early polarization toward Th1 and downregulating Th2 immune responses by stimulating the secretion of tumor necrosis factor-α (TNF-α) and interleukin-6 (IL-6) (43). We found that CD14 expression level was positively correlated with the proportion of Tfh cell and Th2 in GD tissues but not correlated with the proportion of Th1 cell in GD tissues (Figure 1). The involvement of Th1 cell and Th2 cell in the pathogenesis of GD and HT is currently recognized, but its specific mechanism remains to be seen (1) and our findings can bring more information in this area. We have further discovered that there were a total of 93 significantly enriched pathways related to CD14 in GD tissues (Supplementary Table 1). Most of those enriched pathways were immunity-related pathways, suggesting the critical role of CD14 in the development of GD through regulating immune response. Interferon-gamma-mediated signaling pathway, Regulation of toll-like receptor signaling pathway and Positive regulation of interleukin-6 production ranks in the top three, with the greatest relationship with GD. We first discovered that CD14 may mediate the pathogenesis of GD through these pathways.

In conclusion, we, for the first time, demonstrated the significant association between genetic variations in CD14 and GD, and this relationship is more pronounced in female GD patients and GD patients with family history. To further demonstrate the role of CD14 in GD, we conducted a bioinformatics analysis and found that CD14 expression level was positively correlated the proportion of macrophages M1 cell and M1/M2 ratio in GD thyroid tissues. Additionally, CD14 expression level was also positively correlated with the proportion of Tfh cell and Th2 but not correlated with the proportion of Th1 cell in GD tissues. We also found that there were many pathways related to CD14 in GD tissues and most of them were immunity-related. However, the more in-depth mechanism of CD14 in the pathogenesis of GD requires more experimental research. Moreover, to further verify the role of CD14 gene variations in GD, it is necessary to conduct studies with larger sample sizes and more ethnicity.

## Ethics Statement

All patients and healthy controls have signed informed consent. The study was approved by the ethical committees of Jinshan Hospital.

## Author Contributions

XJ was responsible for the experimental design. BW, QY, and QL participated in sample collection. JZ played the guiding role.

### Conflict of Interest Statement

The authors declare that the research was conducted in the absence of any commercial or financial relationships that could be construed as a potential conflict of interest.
